# Sports, culture and tourism in cities along the Beijing-Hangzhou Grand Canal: sustainable development via SWOT analysis and point-axis spatial optimization

**DOI:** 10.3389/fspor.2026.1930614

**Published:** 2026-08-26

**Authors:** Tian Hongda, Zhang Yonghu

**Affiliations:** School of Physical Education, Liaocheng University, Liaocheng, Shandong, China

**Keywords:** Beijing-Hangzhou Grand Canal, canal sports culture, point-axis development model, sports tourism, SWOT analysis

## Abstract

The Beijing-Hangzhou Grand Canal (BHGC) serves as a significant cultural, economic, and ecological corridor in China. The cities situated along the canal possess distinctive sports, cultural, and tourism resources that enhance their prominence within the global canal system. Nevertheless, it also faces challenges, such as the necessity to strengthen the inheritance and utilization of sports culture, enhance the quality of the tourism experience, and deepen the integration of sports culture with tourism (SCT). Moreover, current research lacks a comprehensive review of the resource endowment, developmental advantages, limitations, opportunities, and threats related to SCT in cities along the BHGC. This study utilizes the SWOT analysis framework to evaluate the strengths, weaknesses, opportunities, and threats linked to SCT along the BHGC, based on data derived from literature reviews, field surveys, and expert interviews. In light of these results of SWOT, we recommend leveraging developmental opportunities, such as the establishment of the National Cultural (GCNC) Parks along the BHGC, the rising disposable income of residents along the canal, and the successful bid for World Heritage status, to advance regional planning. Simultaneously, we strive to mitigate risks such as tourist diversion across various tourism markets, inadequate coordinated growth, and a scarcity of specialized professionals. Our approach involves leveraging internal and resource strengths to implement strategies encompassing sports tourism demonstration bases, comprehensive development, collaborative marketing, and integrated services. Furthermore, building on these analysis results, we have devised a point-axis development framework termed “one axis, three segments, and multiple nodes,” drawing on cultural route and point-axis model theories to inform spatial planning. Future research will concentrate on improving the inclusion of tourists and residents in the sample and refining qualitative research methods prior to quantitative analysis. This paper offers theoretical underpinning and practical insights for BHGC cities to enhance their sustainable cultural tourism.

## 1 Introduction

Sports tourism, representing a distinctive convergence of the sports and tourism sectors, has garnered significant attention and popularity as a specialized form of tourism since the 1990s. It is increasingly recognized as a crucial catalyst for the sustainable socioeconomic development of various regions (1, 2). For example, the United States leads the world in the number of sports tourists, with expenditures related to sports travel contributing notably to employment opportunities and fiscal revenues (3). In Europe, particularly in Germany and Spain, these countries account for 57% of the growth in the global sports tourism market (4). Additionally, the rapid economic expansion in China has made outdoor recreation and sports tourism the most preferred activities among the populace (5). The BHGC in China, recognized as a World Cultural Heritage site, is rich in historical and cultural resources, as well as distinctive natural landscapes, which offer significant advantages for the development of sports tourism. Since its successful inscription on the World Heritage List in 2014, research into canal culture and the expansion of its tourism industry has gained considerable attention (6). This interest has accelerated the commercialization of canal culture and created new development opportunities for cities situated along the canal. He and Wu proposed strategies for developing sports tourism resources while ensuring ecological environmental protection (7). The rapid growth of sports tourism has profound environmental, economic, and social implications for the sustainable development of tourist destinations. Consequently, the intrinsic relationship between sports tourism and sustainable development has increasingly captured the interest of scholars and policymakers (8).

In recent years, significant advancements have been achieved in the field of sports tourism research. Chen et al. systematically examined the factors that influence sports tourists' decision-making through meta-analysis, thereby offering a comprehensive perspective on sports tourism consumption behavior (9). Regarding the assessment of sustainable development, Dong et al. performed a SWOT analysis of sports tourism development, using Hainan as a case study, which provides methodological insights for regional sports tourism planning (2). Additionally, Xing et al. investigated the optimization of sports tourism marketing strategies within the context of the SWOT framework (10). Nevertheless, existing studies exhibit several limitations. For instance, most research relies on cross-sectional data and lacks longitudinal tracking studies, which complicates the understanding of the dynamic evolution of sports tourism impacts. Geographically, prior studies predominantly focus on North America, while relevant research on sustainable sports tourism in Asian contexts, particularly in China, remains relatively scarce (11). More importantly, sports tourism is highly sensitive to external environmental changes (12). Unconventional emergencies, such as COVID-19, have inflicted severe damage and extensive impacts on national and regional sports tourism, as well as inbound and outbound tourism markets (13). Due to its high vulnerability to public emergencies and its industrial reliance on concentrated consumer flows, sports tourism has become one of the sectors most drastically affected by the pandemic, resulting in significant negative repercussions for the global tourism market. These successive chain reactions have severely impaired the overall operational efficiency and profitability of China's tourism industry. Nonetheless, opportunities arise alongside crises. It is imperative to conduct in-depth research into the development prospects, constraints, and new sustainable development models of sports tourism in the post-pandemic era.

To address the significant negative effects of the COVID-19 pandemic and promote sustainable development in SCT along the BHGC in canal cities, the Chinese government has introduced a series of policy documents to bolster industrial progress. The “*Outline for the Development of National Tourism and Leisure* (2022–2030)” (14) advocates for the generation of sports-infused tourism products, augmentation of sports tourism services, and the “*Development Plan for the Outdoor Sports Industry* (2022–2025)” (15), led by the General Administration of Sport of China and seven other ministries, focuses on the entire outdoor sports industry chain. It emphasizes the amalgamation of outdoor sports with tourism, the cultivation of premium sports tourism routes and events, and the optimization of land utilization through fiscal and tax support measures. Furthermore, the “*Opinions on Unleashing Sports Consumption Potential and Further Promoting the High-Quality Development of the Sports Industry*” [Document No. (2025) 31] issued by the General Office of the State Council (16) explicitly advocates for broadening sports consumption opportunities, organizing the National Outdoor Sports Industry Development Conference, establishing top-tier boutique outdoor sports routes, and propelling the comprehensive integration of sports and tourism. The implementation of favorable policies has positioned sports tourism as a catalyst for boosting domestic demand, facilitating industrial integration, and driving high-quality regional economic growth.

Consequently, it is urgent to conduct a comprehensive evaluation of sports-related cultural tourism resources in cities along the BHGC, and construct sustainable development strategies and innovative models for SCT along the BHGC in canal cities in the post-pandemic era. To address the above research gaps and practical demands, we focus on three key research questions: (1) What are the SCT resource endowment characteristics and current development status along the BHGC; (2) What are the internal strengths and weaknesses, as well as external opportunities and threats for SCT along the BHGC development, and what should be the strategic direction? (3) How can we build a matching spatial development pattern and feasible implementation paths from the dual perspectives of industrial integration and spatial optimization to promote the high quality and sustainable development of SCT along the BHGC in canal cities?

## 2 Methods

### 2.1 Research objects and investigation sites

**Figure 1:**
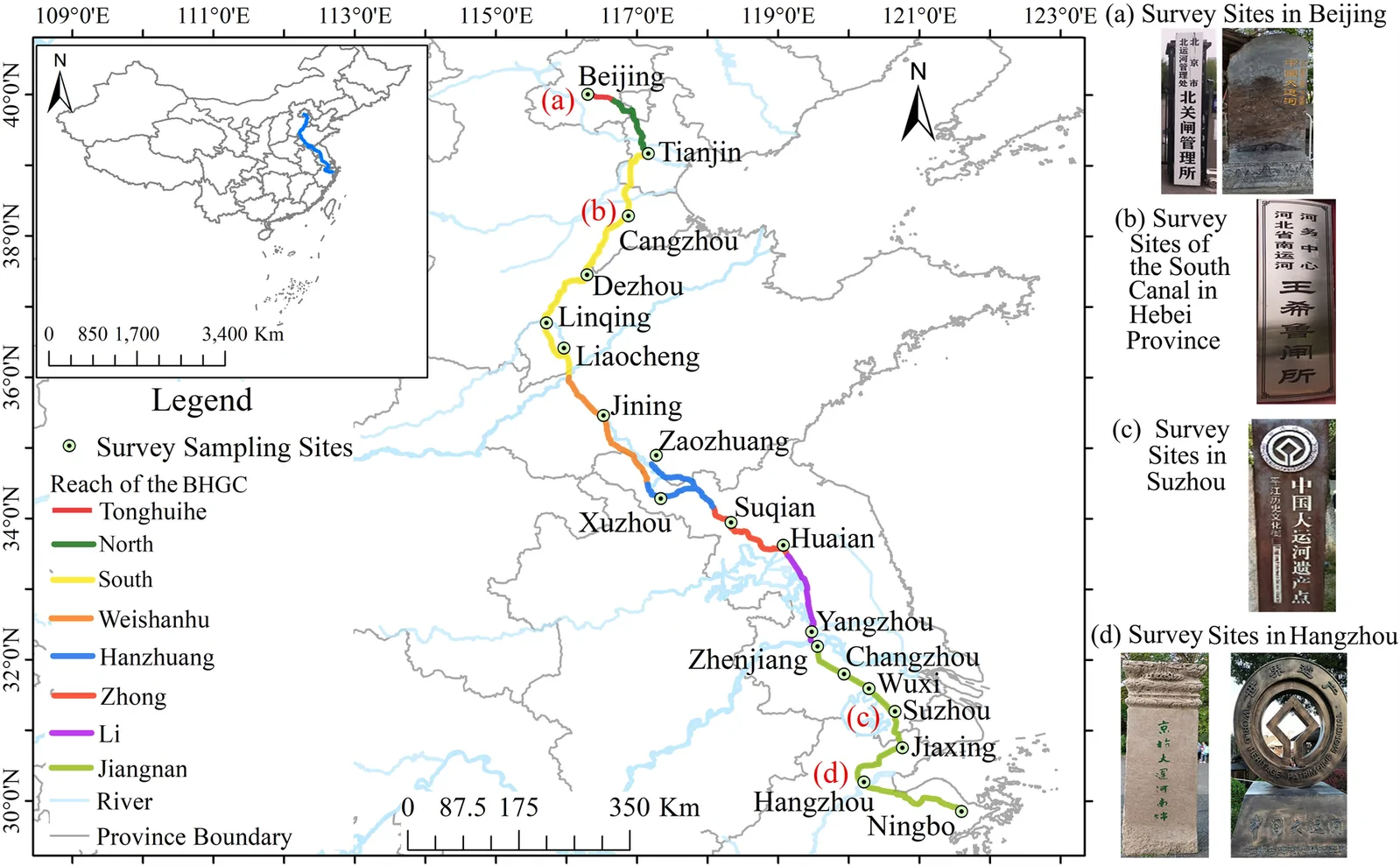
Spatial distribution of reaches and surveyed cities along the BHGC in China.

This study examines the cultural tourism resources related to sports and the current development status of cities along the BHGC in China, a heritage from the Ming and Qing dynasties. Eighteen cities, districts, and counties along the canal have been chosen for analysis, spanning from north to south. These locations include Tongzhou District in Beijing, Wuqing District in Tianjin, Cangzhou, Dezhou, Liaocheng, Jining, Zaozhuang, Xuzhou, Suqian, Huai'an, Yangzhou, Zhenjiang, Changzhou, Wuxi, Suzhou, Jiaxing, Hangzhou, and Ningbo (Figure 1). Survey respondents consist of staff from sports and cultural departments, canal administrative authorities, and relevant management personnel in these regions. The empirical research assesses resource endowment and characteristics, performs a SWOT analysis, and investigates development models of SCT along the BHGC in canal cities.

### 2.2 Progressive analytical approach

We tackle these questions by a progressive approach: starting from fundamental cognition, to strategized diagnosis and finally modeling. We build on this framework an integrated analysis combining theoretical analysis and empirical validation. First, review relevant literature, research advances, and gaps to establish the theory and the main issues in this paper. Field studies and interviews to obtain firsthand information on resource status, developmental challenges and practical needs and provide evidence to support the hypothesis and facts. Then, use SWOT analysis to assess industrial development situation and develop development strategies (SO, WO, ST, WT), which diagnoses development situation and recommends strategies for future analyses. Finally, using SWOT analysis with Pole-Axis system theory, develop sustainable spatial development model and countermeasures and recommendations for sustainable development.

#### 2.2.1 Literature and fieldwork

This paper summarizes and organized literature on tourism, sports tourism, sports industry and canal sports culture from CNKI and Chaoxing Library during 2016 to 2025, statistical yearbooks for cities along the Grand Canal 2023–2025, and a logic of induction, deduction and integration were used to organize and analyze the collected data and materials. Based on these results, we conducted fieldwork in major cities along the BHGC north to south in four provinces and two municipalities along the canal from north to south [some sites are shown in Figures 1(a)–(d)]. This study consolidates previous research findings, resources, and limitations while pinpointing deficiencies in existing studies and highlighting the practical significance of the current research.

#### 2.2.2 Interviews

**Figure 2:**
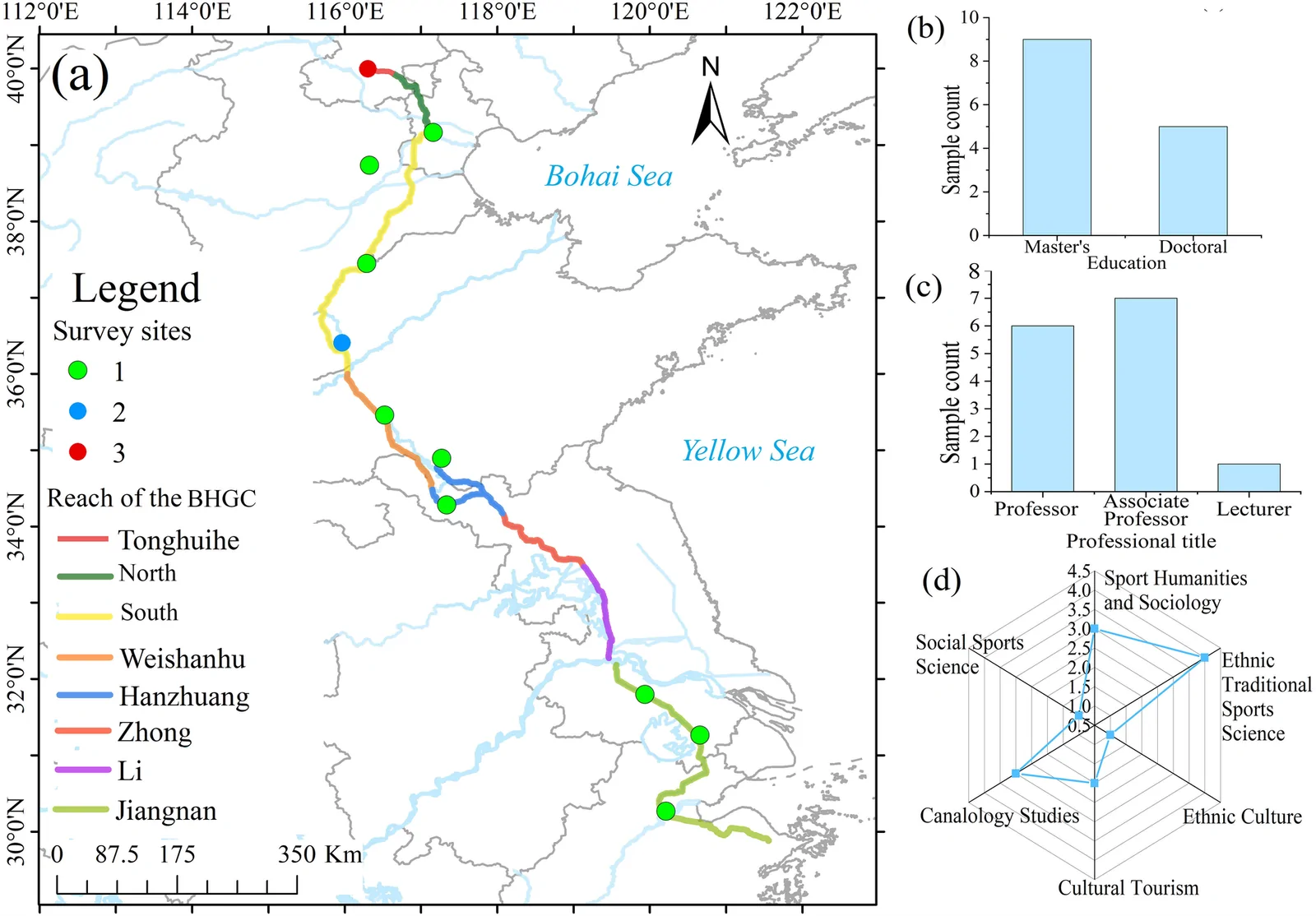
Statistics of interviewed experts along the BHGC in China.

This study employs a comprehensive interview methodology that includes structured, semi-structured, and unstructured interviews to gather data, with each session lasting between 60 and 90 min. The interviews encompassed field visits to destinations of SCT along the BHGC. The survey was conducted in two phases April–June 2024 (Nord and Middle Canal) and September 2024–January 2025 (Süd Canal, Jiangnan Canal and Eastern Zhejiang Canal), the detailed information was presented in Section 2 of the Supplementary Material. There were 67 interviewees including 14 industry experts and scholars (Figure 2), and 53 managers and scenic area operators. These experts were affiliated with the General Administration of Sport of China, sports, and tourism bureaus from four provinces and two municipalities along the BHGC, as well as universities located in canal cities. The interviews were conducted through on-site visits, seminars, and telephone communications and the relevant interview outline was provided in Section 3.1 of the Supplementary Material. This study employs non-invasive consultative interviews in social science, avoiding human sample collection, physiological testing, or psychological intervention. Participants include industry practitioners, university scholars, and public institution staff discussing industrial policies. The approach meets ethical criteria for social science interviews at the university. Refer to Section 4 of the Supplementary Material for more ethical protocols on expert interviews.

#### 2.2.3 SWOT analysis and the extraction, screening and classification of its elements

**Figure 3:**
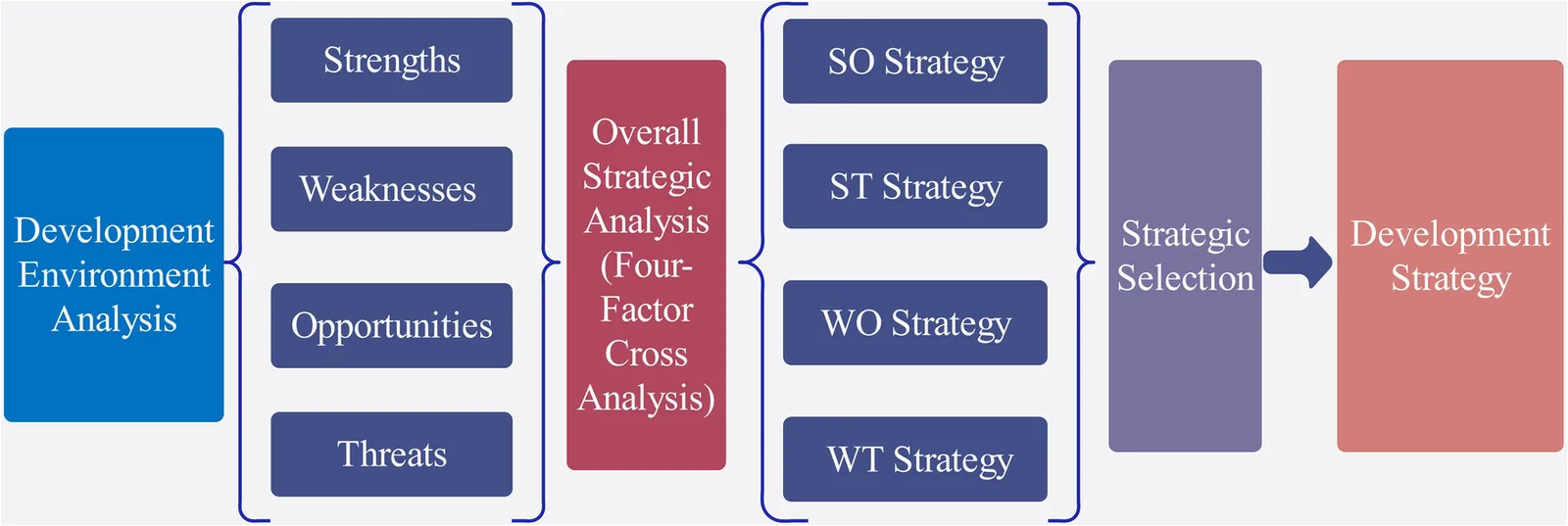
SWOT analysis factors and process.

SWOT analysis is a strategic planning technique, and that primarily examines the conditions within internal and external competitive environments, assesses an enterprise's strategic position by identifying internal strengths and weaknesses, as well as external opportunities and threats, and establishes future development goals (17, 18). Since the 1990s, SWOT analysis has been extensively utilized in various industries, including tourism planning and management in China (19). The SWOT analysis employed in this study encompasses four dimensions consisting of internal and external factors, as shown in Figure 3. Using this method, we perform a matching analysis of various influencing factors to thoroughly diagnose the existing issues in SCT along the BHGC in China.

To mitigate subjective bias and guarantee the authenticity, representativeness and validity of internal and external factors for SWOT analysis, this study identifies all SWOT elements (strengths “S”, weaknesses “W”, opportunities “O”, threats “T”) via multi-source triangulation extraction, two-round expert consensus screening and cross-data validity testing. Detailed extraction, screening and classification approaches for SWOT elements are shown in Section 3.2 of the Supplementary Material.

However, this paper mainly focuses on qualitative analysis and the construction of the pole-axis spatial development model. In subsequent quantitative studies, quantitative methods such as the Analytic Hierarchy Process (AHP) will be introduced to assign weights to each core SWOT factor, so as to realize quantitative strategic evaluation.

#### 2.2.4 Point-axis development model

Based on SWOT analysis strategic goals such as cross-regional coordinated development and spatial layout optimization, we propose point-axis system theory. By combining spatial distribution of SCT resources and economic development status of cities along the canal, we construct a spatial framework of “one main axis, three segments, and multiple nodes” where the overall SWOT strategy goals are translated into practical spatial layout plans that provide a platform for integrated development.

## 3 Results

### 3.1 Endowment and characteristics of SCT along the BHGC resources

#### 3.1.1 Resource endowment of sports cultural tourism

Resource endowment refers to the quantity of producible factors owned and available for utilization by an economic entity (20). In this study, the production factors associated with SCT along the BHGC primarily include natural resources, geographical environments, cultural resources, historical sites along the canal, traditional sports resources, canal sports parks, canal sports venues, and canal sports events. We systematically sorted out the sport's cultural tourism resources of 18 cities across 4 provinces and 2 municipalities along the Along the BHGC in China shown in Figure 1.

Our statistics indicate that following the successful inscription of the BHGC on the World Heritage List in 2014, a phase of conservation and development occurred from 2016 to 2020, during which only 3 iconic Grand Canal-themed (GC-themed) cultural and sports facilities underwent upgrades or were completed. During the next peak construction phase (2021–2023), 17 new benchmark projects and cultural and sports facilities being upgraded or completed. This timeline aligns entirely with the unveiling and execution of the GCNC Parks Construction and Protection Plan. The Jiangsu section is the densest project area, the “golden waterway” and the main heritage area of BHGC. Between 2024 and 2025, following the recovery of cultural tourism market after the Pandemic, six new GC-themed parks and supporting facilities will be constructed, although construction volumes and scale have slowed considerably. The GC-themed parks and supporting facilities span 18 cities along BHGC (Figure 1). Supporting cultural and sports facilities include fitness trails with 600 km length, 327 multi-purpose sports (basketball, badminton, tennis, etc.), 12 national fitness centers, 8 water sports bases and 17 zones for intangible cultural heritage sports. These waterfront sports parks combine national fitness, professional events, leisure sightseeing, and a Grand Canal cultural experience. Table 1 summarizes the annual statistics on typical GC-themed parks and supporting cultural and sports facilities in each section of BHGC.

**Table 1:** Statistical of the annual number of iconic GC-themed parks and cultural & sports facilities in different sections of the BHGC.

Sections	2016	2017	2018	2019	2020	2021	2022	2023	2024	2025	*Σ*
Tonghuihe	—	—	—	—	—	1	1	1	1	—	4
North GC	—	—	—	—	—	—	1	—	—	—	1
South GC	—	—	1	—	—	—	1	2	—	—	4
Weishanhu GC	—	—	—	—	—	1	—	1	1	1	4
Zhong & Li GC	—	1	—	—	1	2	2	1	1	—	8
Jiangnan GC	—	—	—	—	—	2	1	—	1	1	5
Σ	0	1	1	0	1	6	6	5	4	2	26

#### 3.1.2 Characteristics of SCT along the BHGC resources

The BHGC of China has been used for over 2,500 years and is the longest canal in the world. Cities along the canal have preserved many canals cultural and folk cultural assets (include indigenous traditions and festivals). Cities along the canal have also preserved many tangible cultural sites (historic towns, stone bridges, pagodas, and sluices). From a production factors perspective, sports and cultural resources along the BHGC have distinct characteristics (i.e., regional ecological landscapes, resource endowments, preserved culture). A variety of regional sports and cultural ecosystem has emerged around canal cities with unique features. The Public Fitness Corridor Project of the BHGC in Yangzhou, completed from March 2019 to October 2021, focuses on ecological protection by repurposing idle land resources like scattered green spaces, tailored to local conditions. This initiative establishes a unique canal cultural and tourism zone that combines ancient canal sightseeing, sports, cultural experiences, leisure activities, and national fitness, benefiting both locals and tourists. The enhancement of these sports, cultural, and ecological assets align with the sustainable development principles of openness, innovation, coordination, green development, and resource sharing outlined in the *National Fitness Implementation Plan and the Healthy China 2030 Outline* (21). This endeavor is poised to significantly promote public health initiatives and enhance the overall physical well-being and health status of individuals.

Our results indicate that SCT along the BHGC resources in cities along the canal can be classified according to three main categories:

(1) Completeness. Sports events, modern leisure and entertainment, festivals, folk performances, landscape sightseeing. For example, Supplementary Table S1 sorts out the abundant and complete characteristics and types of SCT along the BHGC resources along each section of the BHGC.
(2) Openness. Various SCT along the BHGC resources are integrated into different sections of canals, providing constant creative inspiration for development, design and innovation of SCT along the BHGC and related cultural and creative products.
(3) Dynamics. Combination development in multiple scales and models satisfies the different needs of tourists and market, supported by three important elements, i.e., capital market, information technology, and professional talent.

### 3.2 SWOT analysis of urban SCT along the BHGC

#### 3.2.1 Strength

##### 3.2.1.1 Advantages of folk sports tourism resources

Embedded in the historical folk sports culture of urban agglomerations along the BHGC are diverse traditional folk SCT resources encompassing singing, dancing, instrumental performances, competitive games, and fitness activities. As illustrated by the characteristics of SCT along the BHGC resources listed in Supplementary Table S1. These resources exhibit regional diversity and widespread popularity. Examples include dragon and lion dances, lantern shows, lantern appreciation, stilt walking, variety shows, spring outings, kite flying, dragon boat races, mountain climbing, and traditional boat boxing performances in Suzhou during the Dragon Boat Festival and Mid-Autumn Festival. Suzhou maintains the boxing boat tradition during the Dragon Boat Festival and the Mid-Autumn Festival. Ethnic minority groups possess rich sports cultural resources within their traditional festivals. For instance, the Dong people in Beijing celebrate the Wrestling Festival, the Hui people in Cangzhou host the Lantern Festival Wushu Gathering, and the Miao people in the Jiangnan region partake in swing festivals and grand dragon boat events, as shown by the types of sports tourism resources listed in Supplementary Table S1. Notably, the Jiangsu-Zhejiang section of the BHGC is renowned for its distinctive dragon boat races. The geographical development of dragon boat sports in China has evolved from south to north. Large-scale dragon boat competitions in northern China began in the early 21st century, with the Shandong section of the canal hosting events on canal waterways, lakes, and other water areas, attracting an increasing number of participants annually (22). Such folk sports represent a unique competitive cultural form passed down through generations, marked by distinct characteristics of collectivity, heritage and standardization.

(1) Collectivity is reflected in coordinated efforts between government guidance and non-governmental organizations, which rally public support via digital networks;
(2) Heritage stems from profound historical heritage, sound inheritance systems and rich spiritual connotations;
(3) Standardization manifests in clear organizational codes, coordinated operation mechanisms and mature management norms (22).

Most importantly, these SCT along the BHGC resources can popularize educational contents related to public morality and folk culture among the general public.

##### 3.2.1.2 Policy advantages

Since the BHGC was inscribed on the World Heritage List in 2014, a series of policies integrating canal' SCT have been successively issued by the central government and local authorities. These policies have provided unprecedented support for the innovation and sustainable development of SCT along the BHGC in canal cities. For instance, the Guiding Opinions on Vigorously Developing Sports Tourism, jointly issued by the former China National Tourism Administration and the General Administration of Sport of China in December 2016, elucidates the mutual reinforcement and complementation between cultural tourism and sports tourism. The Master Plan for Tourism of the BHGC, initially published in 2013 and revised in 2015, approved the waterway regulation project for Class III waterways in the Jiangsu-Zhejiang section of the BHGC (23). The 13th Five-Year Plan in 2016 included strategies for the cultural belt protection and navigation utilization of the BHGC, covering Tongzhou District of Beijing, Wuqing District of Tianjin, and Xianghe County of Hebei Province. Canal tourist navigation commenced in 2018, followed by the operationalization of passenger and cargo navigation in 2020, significantly boosting the integrated cultural, tourism, and sports industry of the BHGC (24).

Meanwhile, the central government and provincial and municipal authorities along the BHGC successively developed and executed policies related to SCT along the BHGC during 2016–2021, as shown in Supplementary Table S2. For instance, Jiangsu Province released key documents from 2022 to 2025, such as *Implementation Plan for World-Class Cultural Heritage Tourism Corridor of BHGC* (25), *Project on Heritage Protection and Sustainable Development of Canal Cities* (26), *Protection and Inheritance of Grand Canal Cultural Heritage* (27), and *Plan for Integrated Development of SCT along the BHGC* (28). These policies include regional canal tourism systems, ecological conservation, restoration of old waterways, preservation of heritage sites, tourism growth, digital cloud platforms, all designed to shape collectively the cultural and tourism identity of the “Millennium Grand Canal”. In addition, Expo of BHGC, unique cultural, tourism and sports festivals, interregional coordination's, social capital initiatives have been set up, demonstrating the recognition, by the national authorities and municipalities along BHGC, of the potential and advantages of the integrated canal culture, tourism and sports sector.

#### 3.2.2 Weakness

##### 3.2.2.1 The inheritance and utilization of sports culture still need to be strengthened

The sports culture of canal cities emerges and develops from the integration of unique regional cultures within riparian areas. Over 1,200 cultural relics and historic sites are distributed along various segments of the BHGC as shown in Figure 1, encompassing water conservancy remnants, ancient canal waterways, and historic towns (29). Since the initiation of the national Intangible Cultural Heritage Protection Project in the early 21st century, traditional ethnic sports have undertaken the crucial role of preserving and promoting exemplary traditional Chinese culture. Governments at all levels have facilitated their transformation and innovation in artistic expression, educational application, and market operation, thereby achieving two objectives, i.e., advancing traditional culture and overcoming the challenges of cultural inheritance (30).

Currently, the inheritance model for sports-related intangible cultural heritage of the BHGC relies mostly on static museum display and short festival display. For example, our survey results indicate that digital dissemination is fragmented: multimedia short films about Linqing Canal Boatmen's Chants and Jining Canal Dragon Lantern Dances are often not archived sustainably. Moreover, unique folk sports such as Tianjin Canal Stilt Walking and Linhuan “Two-Ghost Wrestling” in Anhui are unable to have permanent performance facilities and support for young talent. The transmission of these traditions is limited to small family groups and elderly practitioners, resulting in little public participation. Therefore, we urgently need to investigate the cultural and spiritual significance of the BHGC and improve the development effectiveness of tourism resources for sports culture of BHGC.

##### 3.2.2.2 The experience quality of SCT along the BHGC needs to be improved

Since its inclusion on the World Heritage List, the BHGC has developed its cultural significance. The Eastern Route of the South-North Water Diversion Project has further strengthened this influence by water transport. Cities along the canal are now actively trying to revitalize old canal waterways, recreational facilities, cultural centers, and transport infrastructure. Although many resources are available for integrated SCT along the BHGC projects, they face industrial challenges such as superficial project construction based on aesthetics, lack of diversity in industrial formats, low value products, low profitability and low revenue generation. Transformation is needed for SCT along the BHGC development along the canal from superficial appearances to intrinsic quality (31).

Additionally, cultural and creative products related to sports events are homogenized. There is limited support from local culture, tourism and sports products. Sports leisure programs should be richer and more diverse. Many sports leisure programs are not able to fully exploit their cultural value or generate enough cultural value. For example, attractions along the Shandong and Jiangsu sections of the BHGC offer traditional activities like hiking and standard boat tours, and overlook unique experiences related to canal water conservation culture and grain transportation history. This leads to low tourist engagement and poor immersive experiences. The Dezhou segment of the BHGC emphasize tea culture but lacks experiential activities related to canal dock culture and old water transportation labor scenes. Local authorities must develop and refine immersive interactive programs highlighting regional BHGC characteristics.

#### 3.2.3 Opportunity

##### 3.2.3.1 The successful inscription of the BHGC on the world heritage list promotes the upgrading of regional planning

Since 2014, cities along the BHGC have improved production factors like canal resources, labor, institutional structures, technology, and other factors. This development has maximized allocation, quality, and improved industries related to SCT along the BHGC resources. Beijing has planned and established 7 cultural zones along the BHGC such as Baifu spring, Summer Palace, Wanshou Temple, Shichahai-Yu River area, Tonghui River Corridor, old Tongzhou City and Shandong Province has also focused on developing complexes like BHGC's Cultural Corridor in Wucheng County of Dezhou City, canal characteristic towns in Liaocheng City, national fitness and tourism trails, waterways. All these activities emphasize the essence of canal grain transport culture and Qilu culture (32).

Local governments along the BHGC prioritize urban landscape planning and ecological environment protection. *Planning for Urban Landscape Improvement on Both Banks of Hangzhou Section of the BHGC* (33) introduces “Three Waters” development strategy encompassing water culture, water-based cities and water-themed creativity. By 2025 Hangzhou section will become national ecological corridor supporting Beautiful China construction. For example, in 2016, *Xuzhou City implemented the Heritage Conservation Plan for Xuzhou Section of the BHGC* (34), and *Suzhou City issued the Rules for Territorial Space Control in the Core Control Zone of Suzhou Section of the BHGC* (35). These policies have reduced structural pollution in urban zones and surrounding regions by upgrading sustainable transport system and gradually moving polluting enterprises from old urban areas. Surveys, excavations, identification of the BHGC related intangible cultural heritage and selection and protection of representative inheritors have been undertaken. Overall, cities along the canal have implemented a variety of tailored governance and development measures to improve the natural ecology, social development environment and tourism value of the old BHGC. These measures have also increased the availability of SCT along the BHGC resources, setting up a canal landscape belt of leisure, fitness and experiential functions, contributing to SCT along the BHGC.

##### 3.2.3.2 Historic development opportunities brought by the construction of BHGC national cultural park

The Plan for National Culture Parks for the Great Wall, the BHGC and the Long March (36) outlines the cultural significance of the Cultural Belt of the BHGC, Long March, and Great Wall. It outlines a practical approach to preserve, rejuvenate, and perpetuate the sports cultural legacy of the BHGC, and promotes the amalgamation of cultural, tourism and sports sectors. This cultural initiative has opened new opportunities for cultural, tourism and sports industries along the Cultural Belt of the BHGC. Zhejiang Province has developed several implementation strategies for National Culture Parks along the Cultural Belt of the BHGC, based on the first trials. Sports complexes such as the Cultural Belt of the BHGC Asian Games Park and the Cultural Belt of the BHGC Central Park in Gongshu District, Hangzhou (as shown in Supplementary Table S3) promote national fitness. Combining Asian Games heritage, the BHGC legacy and various sports components, they include stadiums, sports fields, commercial facilities and a wide range of public services. During the Hangzhou Asian Games, they host important competitions such as table tennis and field hockey.

Seven national culture park projects along the Hongqiao section of the Canal of Tianjin aim to improve canal dock cruise services and historic cultural tourism areas. Utilizing the resources of the BHGC and the intangible cultural heritage of Wuqiao and Nanpi, Cangzhou City integrated existing projects such as canal intangible cultural heritage exhibition halls and riverside landscape belts to complete the park construction in 2022. The sports culture of the Canal could be reproduced culturally and can be turned into a wealth of integrated SCT resources along the BHGC. The establishment of the BHGC National Culture Parks by riparian cities has been implemented significant decisions and directions of Party Central Committee and State Council to promote SCT sustainable development along the BHGC. This initiative is a new starting point for national culture park development. All provinces and municipalities along the BHGC Cultural Corridor have set up regular cross-regional joint meeting system to overcome the previous development model characterized by fragmentation of market, isolated regional operations and limited local development position. This system sets up a collaborative system with clearly defined rights and responsibility to develop and protect intellectual property rights for cross-regional sports events.

##### 3.2.3.3 Increasing disposable income to drive the growth of sports tourism consumption

**Figure 4:**
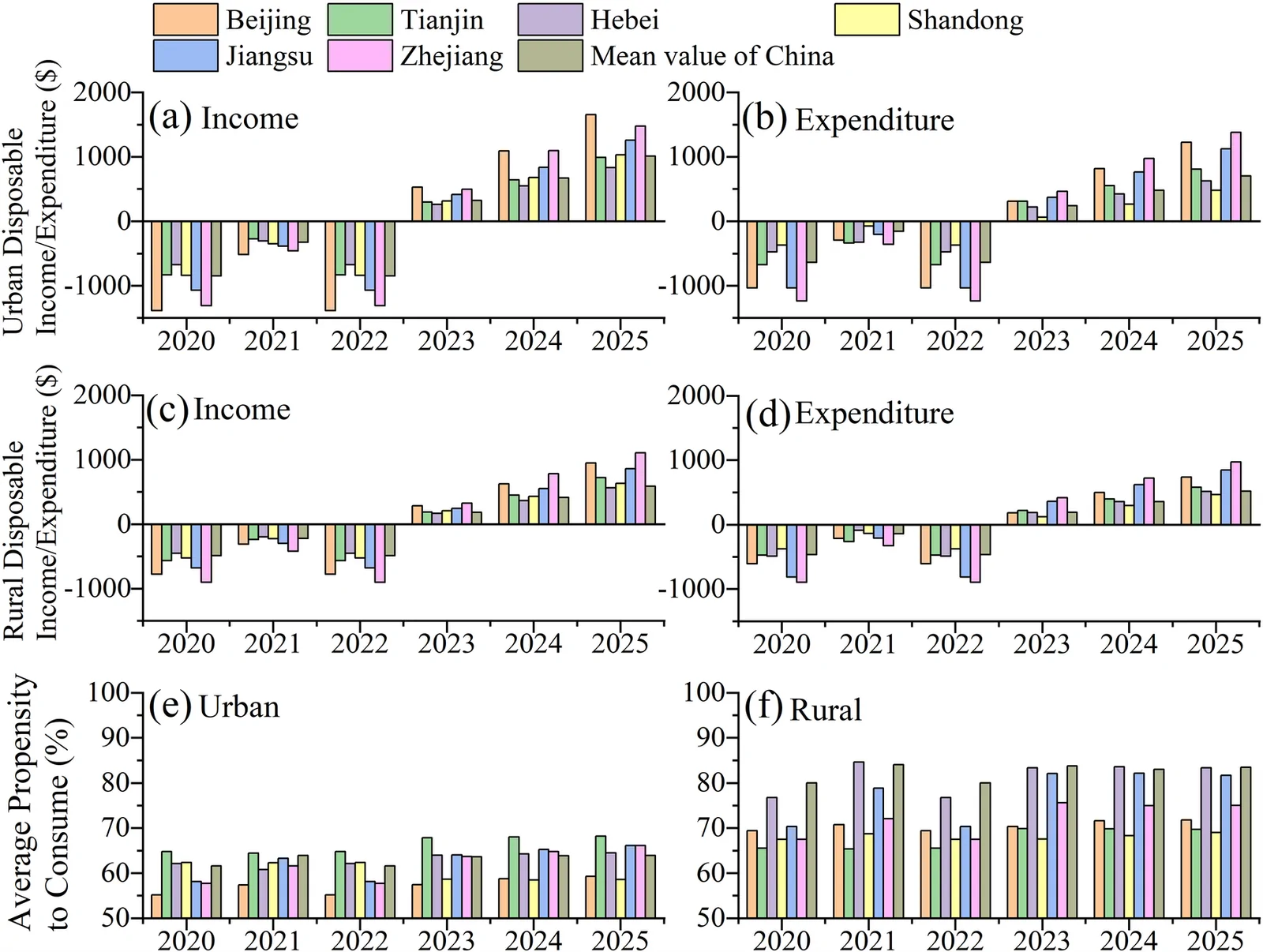
Spatial distribution map of per capita income and consumption expenditure as well as average propensity to consume in provinces and cities along the BHGC.

The areas along the BHGC have seen rapid development and significant improvement in living standards of urban residents. The rural living conditions have also seen remarkable improvement with the transfer of surplus rural labor to non-agricultural sectors increasing steadily. According to FAO criteria, a region is rich when its per capita GDP surpasses a given USD, Engel's coefficient falls between 30% and 40% and culture consumes around 23% of expenditure. The anomalies of each variable relative to the multi-year average from 2020 to 2025 are presented in Figures 4(a)–(d), where positive values indicate figures in Figures (a)–(d) above the average, while negative values represent that below the average (Note: 2020–2024 data are derived from the China Statistical Yearbook, and the 2025 data are preliminary statistical accounting data.). According to Mann-Kendall statistical test, the disposable income and consumption expenditure of urban and rural residents along BHGC have increased sharply Urban residents have Engel's coefficient 34.8% and rural residents have coefficient 37.1%. These data suggest that the socioeconomic advancement of cities along the BHGC has driven the growth of disposable income and increasing demand for high quality SCT services along the BHGC [Figures 4(e),(f)]. This demand indicates significant supply deficit in integrated SCT along the BHGC cities, which has huge untapped market potential.

#### 3.2.4 Threats

##### 3.2.4.1 Tourist diversion caused by diverse tourism markets

The Belt and Road Initiative in China has significantly boosted tourism development, festivals creation, tourism brand establishment, market exploration, tourist involvement, media promotion, international collaboration, and various other endeavors in regions along its routes, fostering comprehensive growth through interconnected corridors. This approach accelerates the advancement of regional cultural, tourism, and sports sectors. While it serves as a leading force in promoting SCT in canal cities, it also poses risks of diverting tourists. Moreover, the SCT's industries in North, Central, and South China, particularly along and near the BHGC, have witnessed rapid expansion. Nevertheless, the excessive standardization of tourism resources has complicated the efforts of canal cities to allure tourists with their combined SCT's attractions.

Globalization and modernization trends in the tourism industry are driving the rapid growth of traditional forms of tourism like outbound travel, coastal tourism, ecotourism, business travel, and vacation travel. Additionally, emerging types of tourism such as desert tourism and aviation sightseeing are also experiencing significant growth. This increasing diversification in tourism offerings is intensifying competition among different sectors, leading to a decline in market share for SCT along the BHGC. As the variety of tourism options expands within national markets, the overall growth in tourist numbers is slowing down, granting tourists greater flexibility in choosing destinations. Consequently, there is a shift in public interest away from SCT along the BHGC, resulting in a notable decrease in visitor numbers. To address these challenges, it is imperative to optimize the utilization of SCT's resources in canal cities to enhance their value, boost brand recognition and market influence at local, national, and international levels, emphasize unique brand attributes and competitive advantages, and improve market competitiveness.

##### 3.2.4.2 Shortage of professional practitioners

SCT along the BHGC are specialists, in which practitioners must have professional guidance, emergency first aid skills, solid foundational sports skills and medical knowledge. This is especially crucial for sports tourism activities such as diving, rock climbing, and outdoor adventure sports. Universities along the BHGC, including Zhejiang University, Soochow University, Changzhou University, Yangzhou University, Jiangsu Normal University, Liaocheng University and Tianjin University, offer courses in sports science, culture industry management, tourism management, etc., but have not established any particular major in sports tourism. Only around 5% of those in sports and tourism are sports related (37). Graduates often have a gap between their sports knowledge and their understanding of the tourism industry, and struggle to combine their practical tourism knowledge with sports knowledge. Also, cooperation between sports, culture, tourism businesses along the BHGC and universities are primarily focused on joint branding and superficial academic interactions, not real resource integration or value creation.

Our statistics indicate that China's SCT sectors face both a very low number of professional talent and a weak market support mechanism. The development of these sectors along the BHGC shows significant lack of both quantity and quality of talent. It is therefore necessary to engage universities, research institutes, professional training centers, SCT's organizations in canal cities to establish a mechanism for industry education integration and collaboration. This should promote training efforts and develop interdisciplinary sports and tourism professionals with sports knowledge, cultural background, and operations and management skills. At the same time, high-end talent is needed in culture and tourism to create a professional pool aligned with integrated development of culture, sports and tourism in the new age.

## 4 Sustainable development strategies for SCT along the BHGC

### 4.1 Sustainable development strategies based on SWOT analysis

#### 4.1.1 SO strategy: rely on internal resource advantages and seize external policy and planning opportunities

**Figure 5:**
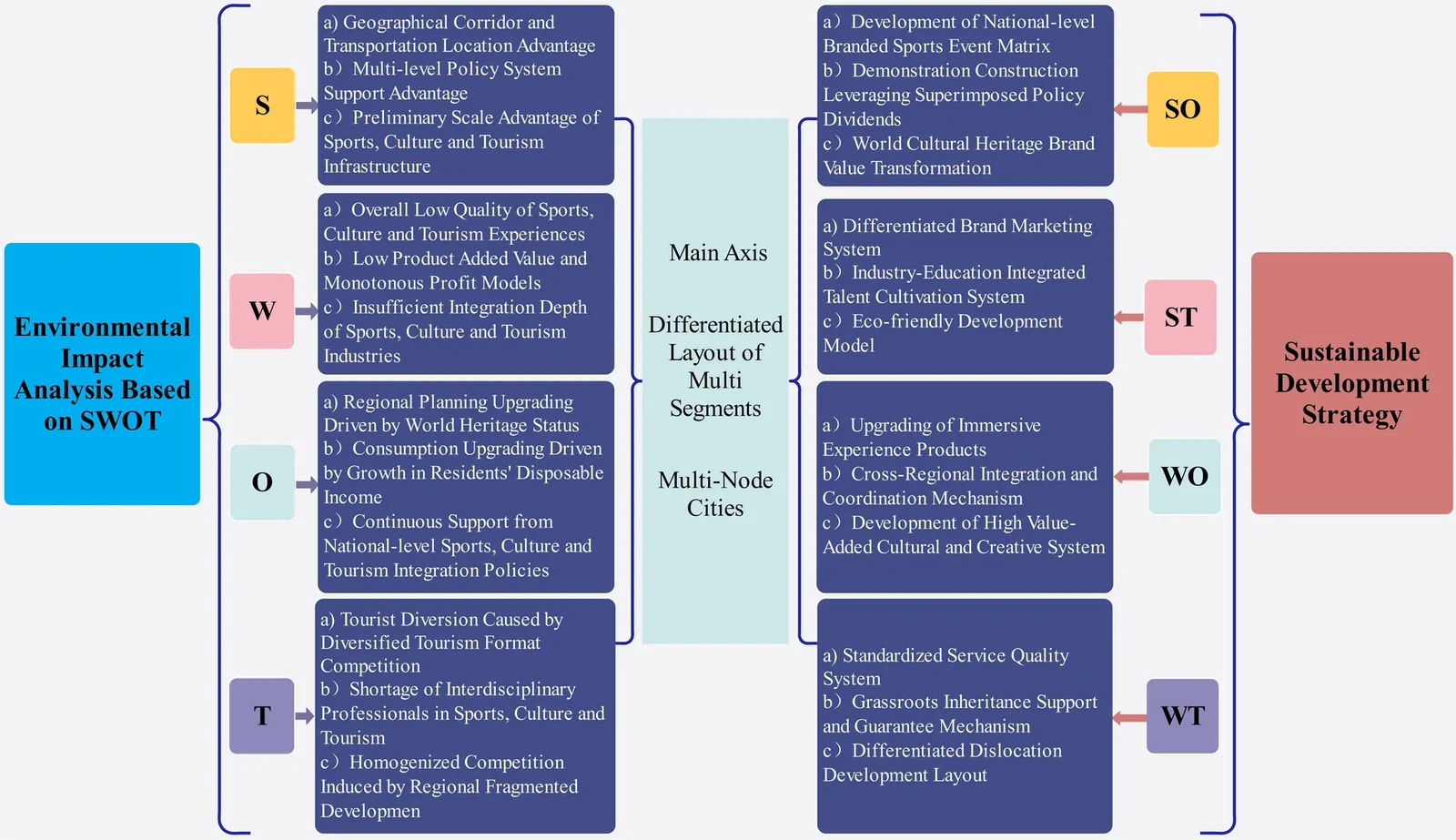
Development strategies for SCT in canal cities based on comparative SWOT analysis.

Integrate intangible cultural heritage elements into canal cities, such as traditional sports acrobatics, folk dances, folk fine arts, and local customs, with historical relics. Develop unique, profitable, and sustainable tourism routes centered on intangible cultural heritage (Figure 5). Implement various performance and interactive programs related to sports-based intangible cultural heritage, and incorporate these heritage elements across all tourism aspects, including dining, lodging, transportation, sightseeing, shopping, recreation, and entertainment. Enhance event planning to boost the integration, visitor engagement, and immersive quality of intangible cultural heritage initiatives, while expanding the outreach of canal cultural and sports tourism offerings.

Furthermore, in the realm of ecological preservation and regional development, it is crucial to thoroughly explore the unique natural resources of the BHGC, including mountains, rivers, forests, farmlands, lakes, grasslands, and wetlands, along with traditional cultural elements. By focusing on canal sports culture, encompassing mountain pursuits, outdoor activities, water sports, aviation endeavors, martial arts, and popular sports events with significant public involvement, the goal is to establish premium integrated industrial zones and cultural corridors that blend culture, sports, and tourism in canal communities. Simultaneously, the focus is on developing distinctive sports activities such as hiking trips, cycling races, marathons, and dragon boat competitions utilizing the green ecological zone of the BHGC, and fostering unique sports event brands like “Walking Along the BHGC,” Dragon Boat Races of BHGC, and rowing tournaments.

The promotion of competition tournaments, national fitness programs, tourism programs and cultural experiences along the canal is aimed at integrating SCT. The 14th Five-Year Plan for Tourism Development issued by the State Council in 2021 (38), and the Guiding Opinions on Vigilance Development of Sports Tourism issued by General Administration of Sport of China in 2016 (39), promote cultural dialogues and cooperations between the regions along the canal and create an industrial environment for sports, culture and tourism.

#### 4.1.2 WO strategy: seize external development opportunities and make up for shortcomings in internal coordination and resource development

By adopting a systematic development approach encompassing comprehensive planning for the entire basin of the BHGC and addressing the diverse tourism demands, our goal is to leverage the SCT assets of canal cities through overlay, fusion, and deep integration. This strategy aims to establish top-tier integrated industrial clusters with synergistic advantages and interconnected systems. Coordinated regional planning studies will be conducted to enhance distinctive SCT projects along the canal, emphasizing their unique regional features and clustering effects. Facilitating the sharing of tourism resources and markets will expand the market reach of SCT along the BHGC, promoting the scientific and sustainable advancement of the canal's SCT sector during its refinement and enhancement phase.

A coordinated development mechanism for SCT between provinces and municipalities along the BHGC will be established for region-wide development based on mutual benefit and win-win outcomes. This includes a standard tourism information platform covering all basins of the BHGC and a common coordinator for development and management. Supporting systems for information release, command and dispatch, risk prevention and control will be developed to connect scenic spots with high-quality tourist routes. Furthermore, cooperation between competent authorities of SCT in canal cities can lead to enhanced links and cross-regional communication. Coordinated short-term, medium-term and long-term development plans, strategies and implementation schemes will be developed. A standard and integrated market system reflecting SCT along the BHGC will be developed for well-organized, logically organized and efficiently operated development layout with primary and secondary functional zones. Development objectives will be focused on complementary functions, structured organization and comprehensive coordination.

#### 4.1.3 ST strategy: relying on the advantages of resource integration to cope with external pressures from market competition

The branding strategy for SCT industries in canal cities should adopt a coordinated development approach emphasizing joint promotion and bundled marketing to effectively consolidate diverse assets. This strategy aims to create new intellectual property representations for SCT along the BHGC, establishing a unified brand with regional distinctiveness, strong recognition, and significant market influence. To support this effort, an innovative communication and promotion framework for SCT along the BHGC must be implemented, leveraging key media platforms such as China Central Television (CCTV). This initiative will involve utilizing government-endorsed media relations to optimize resource allocation, enhance coordination mechanisms, foster partnerships, cultivate positive public interactions, and boost the visibility and market reach of the SCT sector along the BHGC (40). Additionally, new strategies for multi-party media advertising procurement and service models should be developed to improve operational efficiencies.

Moreover, capitalized on the favorable development environment of the national and local regulations, new consumption models for SCT along the BHGC should be cultivated (41). These models include online, customized, experiential, smart, and interactive consumption. Driven by digital developments, new consumption models like cloud tourism, cloud performances, cloud entertainment and live streaming services will be promoted. A wide range of SCT along the BHGC content will be amalgamated into festivals, shows, exhibitions and markets, incorporating sports, cultural and creative goods, holidays and recreation themes, to enhance diversity, dynamism and inclusion of SCT along the BHGC. On the marketing front, bundled pricing strategy based on consumer expenditures will be enforced and a composite tourism offering will be created to appeal to travelers.

An integrated cultural and tourism information platform for the BHGC will be developed as part of the WO strategy. Utilizing artificial intelligence algorithms, real-time tourist data will be analyzed to understand tourist behaviors, assess market trends, and promote sustainable growth in tourism. The collaboration among cultural and tourism businesses in canal cities will be enhanced, fostering long-term partnerships with surrounding scenic attractions. Shared resources from SCT sectors along the BHGC will be leveraged, optimizing the deployment of specialized talents. A comprehensive service network will be established, along with a cross-regional cooperation framework, to effectively target and attract potential consumer groups.

#### 4.1.4 WT strategy: eliminate internal service shortcomings and avoid threats concerning service quality and sustainable development

We emphasized the need to integrate canal sports culture into the current public cultural service system to improve the infrastructure supporting SCT along the BHGC. We aim to improve service efficiency, provide personalized services to tourists after the outbreak and offer immersive SCT experiences for visitors and local people (42). We will design a smart SCT service system consisting of digital service with various transportation networks, online consultation and payment functions, and unified one card service model for SCT along the BHGC consumption. Canal tourist highway transportation system will also be optimized and restructured following the WO strategy by planning and upgrading high quality tourist highways along the BHGC ecological corridor. Enhancement of the micro-circulation transportation network, namely themed tourist routes, ecological landscape roads, cycling lanes, pedestrian corridors and canal waterways (docks). Special tourist transport routes will efficiently link cultural and eco-tourism resources in towns along the canal. Important elements like itinerary planning, professional services and daily consumption will be integrated in a tourism service model, allowing visitors to access all attractions with one ticket while travelling the journey.

An analysis of tourist consumption preferences is essential for advancing the integrated SCT sector. The findings indicate that utilizing a standardized basin-wide tourism information platform (Sect. 4.1.1), adopting a systematic holistic development approach (Sec. 4.1.2), implementing a regionally interconnected coordinated development model (Sec. 4.1.3), establishing a smart SCT service system, and enhancing the transportation system as discussed in the section will facilitate the formulation of market expansion strategies to meet tourists' experiential demands (43). To accomplish this, it is imperative to enhance the focus and expertise of frontline staff and administrators. Moreover, setting up training facilities and professional development institutions for SCT along the BHGC would diversify and broaden inclusive talent development initiatives. These initiatives will augment the pool of professionals specializing in SCT along the BHGC, recruit middle and senior management personnel for the sector, and elevate the overall service standards of the BHGC's sports, culture, and tourism industries. Improving the integrated high-quality cultural and tourism service system along the BHGC would benefit urban and rural residents and tourists. This enhancement will foster refined, scientific, standardized, and sustainable development of SCT along the BHGC.

### 4.2 Development strategies for SCT in canal cities—design of the point-axis model

Point-axis system theory was proposed by Chinese scholar Lu Dadao in 1984 (44). Urban settlements and central cities along the BHGC are called points, while infrastructure belts connected by transportation trunk lines, resources and waterways along the canal are called axes. The regional development model formed by growth poles of upgraded points and axes have siphon effect on production in surrounding areas. Culture and economic components of upgraded points and axes influence neighboring towns and industries, and combine with cultural and economic resources in the peripheral regions to generate new productive forces and new economic growth points. This paper examines value and connotations of SCT along the BHGC using the rich heritage of canal. By optimizing the interconnected sports, cultural and transportation systems mentioned in Sec. 4.1, a systematic and sustainable development model for point-axis model is established and promoted that preserve, use, inheritance and promote canal sports culture.

**Figure 6:**
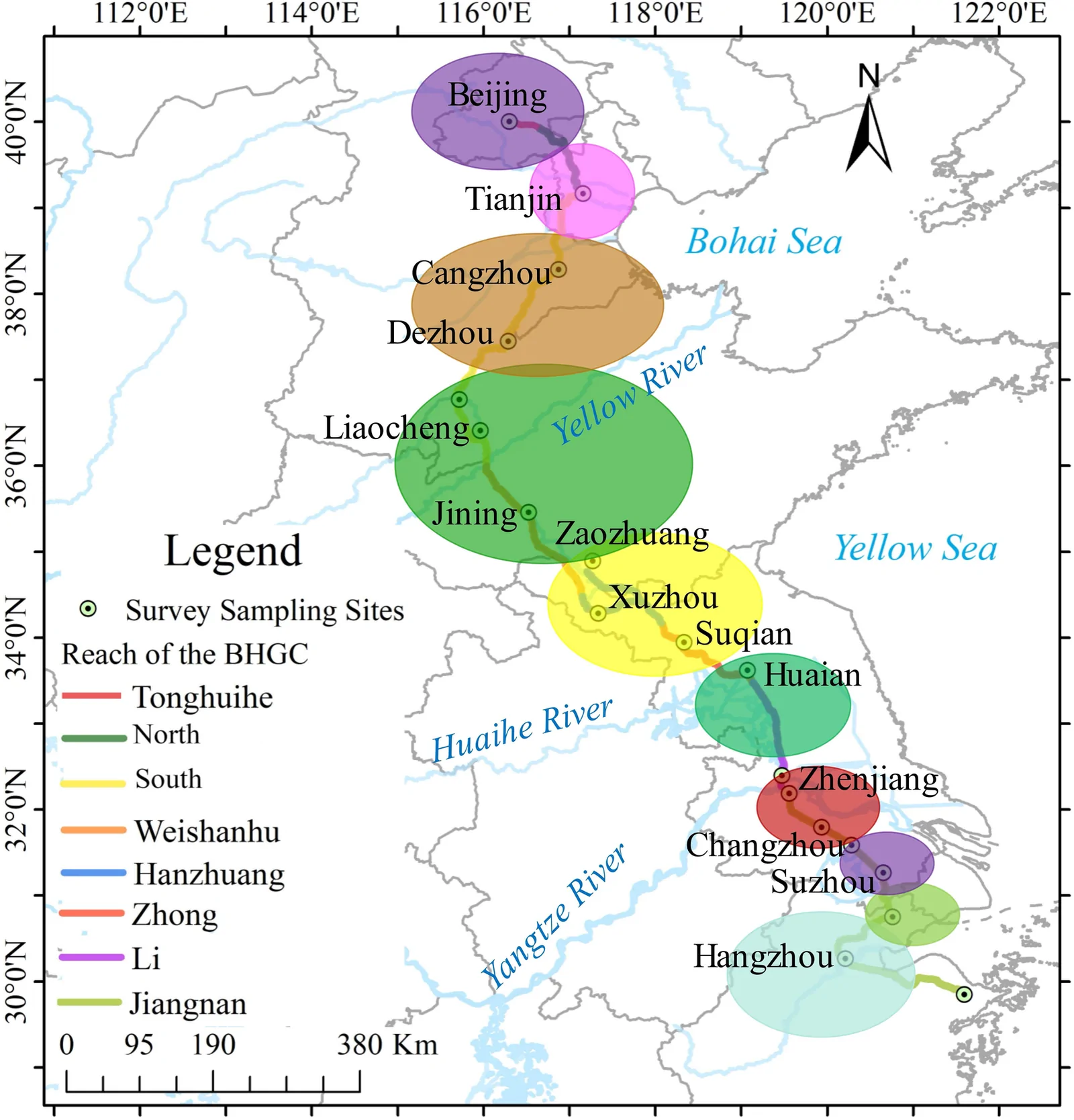
Generalized diagram of point-axis model design for SCT in cities of the BHGC.

Based on the economic development levels of canal cities and the spatial distribution of SCT resources along the BHGC (Figure 6), this research adheres to the principles of scientific conservation and prioritizes planning. It is constructed by aggregating smaller resources for large-scale advantages. It integrates and explores the abundant resources of national historical and cultural cities along the BHGC (cultural centers) and uses their strengths and characteristics to promote SCT development. In addition, by focusing on key river reaches and urban nodes, we establish a central development axis for SCT sectors of canal cities (cultural axis). By focusing on SCT projects as key entry points, we combine natural resources and innovates financial markets and clusters basic sports and cultural resources in order to build a benefit complex and a national demonstration zone.

Driven by the concept of “rivers as lines, cities as pearls, lines stringing pearls, and pearls driving surrounding areas,” this research centers on the BHGC and its neighboring regions. The primary objective is to facilitate coordinated advancement in three divisions: the Beijing-Tianjin-Hebei segment, the Shandong segment, and the Jiangsu-Zhejiang segment of the BHGC. Additionally, the study seeks to establish links among historical and cultural cities situated along the canal, configuring a spatial arrangement of “one main axis, three subsections, and multiple nodes” (see Figure 6). This configuration maximizes the BHGC's pivotal role in connecting areas, constructing spatial networks, and fostering cultural amalgamation and interactions. Consequently, it is crucial to underscore the significant and propulsive function of each “node” in urban SCT, accentuating the connecting role of the “axes” between nodes to propel the comprehensive revitalization of SCT sectors in all canal cities by invigorating surrounding areas.

## 5 Conclusion

Based on China's new development stage, historical missions, and external environment, it is necessary to create new development pattern, focused on domestic circulation and domestic circulation and international circulation mutually strengthening each other. This will help promote high quality and sustainable internal circulation in SCT sectors in canal cities, especially post-pandemic. High quality tourism experience will be the main competitive advantage of scenic spots in the future. Outdoor adventure tourism is also increasingly popular among young people, including nature exploration, ecotourism, wilderness travel, diving, mountaineering etc. This research shown that developing SCT sector in canal cities along the BHGC requires cooperation from different stakeholder.

Developing smart tourism resources that showcase sports-related intangible cultural heritage is essential. Tourism enterprises must adapt their business strategies to changing tourism consumption patterns and travel motivations. They need to accurately understand market demand and fully utilize available resources to align with market trends. Furthermore, tourism academia should delve into the evolution of tourism research over time and across disciplines to offer theoretical backing for the high-quality and sustainable advancement of SCT along the BHGC in the post-pandemic period. However, this study employs qualitative and initial quantitative methodologies. Future research should enlarge the sample size and merge quantitative and qualitative methods for thorough analyses.

Nevertheless, the study sample inadequately represents both tourists and local residents. Analyses of tourism experience quality and consumption demand often focus on the supply side, potentially overlooking actual perceptions and preferences of end users. Future research will involve extensive surveys, interviews, and questionnaires with tourists, operators, and residents to enhance bidirectional supply-demand analysis and sustainable development model application. Initial methods are qualitative and preliminary quantitative; future research will expand to include both quantitative and qualitative approaches.

## Data Availability

The original contributions presented in the study are included in the article/Supplementary Material, further inquiries can be directed to the corresponding author/s.
