# Supplementary material for: Sports, culture and tourism in cities along the Beijing-Hangzhou Grand Canal: sustainable development via SWOT analysis and point-axis spatial optimization
Source: Front Sports Act Living. 2026 Aug 26;8:1930614. doi: 10.3389/fspor.2026.1930614 (PMC13553808; doi:10.3389/fspor.2026.1930614)
Supplement: Supplementary file 1 [file Datasheet1.docx]

**Sports, Culture and Tourism in Cities Along the Beijing-Hangzhou Grand Canal: Sustainable Development via SWOT Analysis and Point-Axis Spatial Optimization**

Tian Hongda^1^，Zhang Yonghu^1*^

^1^ School of Physical Education, Liaocheng University, Liaocheng, Shandong 252000, China

**Corresponding author: E-mail: zhangyonghu@lcu.edu.cn*

1. **The table below is a supplementary appendix added to the main manuscript, with details as follows.**

**Table S1.** Characteristics and Types of Sports Cultural Resources in Different Sections of the BHGC

| River section | Characteristics of Sports Cultural Tourism Resources | Sports Tourism Resource Types |
| --- | --- | --- |
| Tonghui River | Following 2008, a unique 15.8-kilometer waterfront cultural and sports landscape belt was developed along the Tonghui River. This belt features sports parks, pedestrian streets for leisure and sports, and cycling fitness trails. By combining fashion, leisure creativity, and a variety of sports and cultural activities, the belt harmonizes natural and cultural landscapes, sculptures, architecture, and sports facilities into a cohesive entity. | Event Watching  Folk Performance  Festival Participation  Modern Recreation |
| North Grand Canal | Spanning 186 kilometers, the route features integrated sports, cultural, and tourism facilities that blend ecological environmental protection with leisure recreation and fitness entertainment. The construction model emphasizes sports-cultural ecological scenery and local athletic characteristics, promoting resident participation in activities like swimming, dragon boat racing, and cycling. | Event Watching  Folk Performance  Festival Participation |
| South Grand Canal | The waterway spans 509 kilometers and features various cultural and sports facilities such as indoor and outdoor fitness centers, gymnasiums, fitness walking trails, sports parks, fitness plazas and illuminated sports courts. It is totally based on development principle: "Incorporating sports into parks and turning sports facilities into parks." | Folk Performance  Festival Participatory  Modern Recreational |
| Weishanhu Canal | This 140-kilometer waterway supports a range of aquatic sports and cultural activities, including dragon boat racing, yacht cruising, motorboating, swimming, fishing, diving, and water skiing. A speedboat tourism venture was jointly established by local investors at the Weishanhu Sports and Tourism Resort, thereby significantly promoting the development of sports cultural tourism. | Mountain and Lake Leisure  Folk Performance  Modern Recreational |
| Hanzhuang Canal | Covering a total distance of 42.5 kilometers, the Sports Expo City utilizes local ecological resources, including forests, water systems, vegetation, and villages. It also incorporates sports and cultural assets, such as red sports cultural tourism and lantern dance performances, to establish a fitness and sports zone focused on health and wellness. | Mountain and Lake Leisure  Event Watching  Folk Performance  Festival Participatory |
| Zhong Canal | Within Xuzhou territory, a 181-kilometer section of the canal hosts various cultural and sports activities, such as folk sports competitions and regional sports and cultural festivals, engaging the local residents. | Event Watching  Folk Performance  Festival Participatory  Modern Recreational |
| Li Canal | The 170 km long canal houses various cultural heritage sites, including the Zhou Enlai Memorial in Huai’an. Local authorities aim to merge canal sports and cultural activities with sports tourism, leisure services, and themed parks to establish a diverse industrial center focused on Grand Canal sports culture. | Mountain and Lake Leisure  Folk Performance  Festival Participatory |
| Jiangnan Canal | Spanning a total of 323.8 kilometers, the Yangzhou section includes a 15-kilometer fitness corridor adjacent to the historic Grand Canal. This corridor is outfitted with 19 fitness stations and 10 basketball courts, suitable for hosting various activities such as long-distance running, cycling competitions, and cultural events. | Mountain and Lake Leisure  Folk Performance  Festival Participatory  Modern Recreational |

**Table S2.** Statistics of Relevant Policies and Regulations of the State and Provinces and Cities Along the Grand Canal (Sorted by Institution)

| Release date | Document | Institution | Overview of Key Points of Canal Cultural Tourism |
| --- | --- | --- | --- |
| December 2021 | The 14th Five-Year Plan for Tourism Development | State Council | A new national tourism spatial pattern will be established, characterized by point-based radiation, belt-based connection, and network-based coordination, relying on ten projects that include the national cultural parks of the Great Wall, the Grand Canal, the Long March, the Yellow River, and the Silk Road Tourism Belt. |
| July 2021 | 14th Five-Year Implementation Plan for the Protection, Inheritance and Utilization of Grand Canal Culture | National Development and Reform Commission | In 2023, the conservation status of the world cultural and natural heritages associated with the Grand Canal achieved a significant milestone, marked by the establishment of high-quality tourist routes and brands. By 2025, the Grand Canal National Cultural Park is expected to emerge as a vital symbol for promoting Chinese culture globally. |
| September 2020 | Plan for Integrated Development of Grand Canal Culture and Tourism | Ministry of Culture and Tourism, National Development and Reform Commission | Enhance the integrated development of the Grand Canal's cultural and tourism industries by creating high-quality cultural and tourism routes and establishing a series of brands. Promote the cohesive development of public cultural and tourism services along the Grand Canal while reinforcing the safeguards necessary for effective plan implementation. |
| April 2021 | Notice on Holding the 2021 National "Walking Along the Grand Canal" National Fitness Walking Activity | General Administration of Sport, National Development and Reform Commission, Ministry of Culture and Tourism | To implement the Outline for the Protection, Inheritance, and Utilization of Grand Canal Culture, as well as the Construction Plan for the Great Wall, Grand Canal, and Long March National Cultural Parks, it is essential to execute the sports tourism demonstration project. This initiative will involve exploring sports tourism resources along the Grand Canal and establishing distinctive demonstration activity brands for Grand Canal sports tourism. |
| December 2019 | Construction Plan for the Great Wall, Grand Canal and Long March National Cultural Parks | General Offices of the CPC Central Committee and the State Council | Promote the protection, preservation, and utilization of cultural relics and resources through fundamental projects that include exploration and excavation, the development of environmental support facilities, the integration of cultural tourism, and digital reproduction. A coordinated framework will be established for the protection, preservation, and utilization of cultural relics and resources along the Great Wall, the Grand Canal, and the Long March. |
| May 2019 | Outline for the Protection, Inheritance and Utilization of Grand Canal Culture | General Offices of the CPC Central Committee and the State Council | Maximize the utilization of the rich historical and cultural resources of the Grand Canal, while ensuring their protection, inheritance, and effective use. This approach aims to establish the Grand Canal Cultural Belt as a testament to the invaluable heritage bequeathed by our ancestors. |
| April 2023 | Measures for the Administration of National Cultural Industry Demonstration Parks (Bases) | Ministry of Culture and Tourism | Enhance the protection of the Grand Canal heritage, standardize its utilization, and foster comprehensive, coordinated, and sustainable economic and social development along the Grand Canal. |
| December 2019 | Implementation Plan for the Protection, Inheritance and Utilization of the Grand Canal Culture in Beijing | Beijing Municipal People's Government | It establishes medium- and long-term objectives for the protection, preservation, and utilization of Grand Canal culture across three stages: 2025, 2035, and 2050. These objectives encompass various aspects, including cultural relics, ecology, tourism, landscape, and regional coordination. |
| December 2019 | Five-Year Action Plan for the Protection, Inheritance and Utilization of Grand Canal Culture in Beijing (2018–2022) | Beijing Municipal People's Government | The Plan refines, quantifies, specifies, and projects its tasks, which encompass nine major components: establishing the development framework for the Grand Canal Cultural Belt, implementing environmental enhancements along the route, clarifying the historical and cultural context of the Canal, advancing the development of cultural projects, improving tourism and leisure functions, fostering cross-regional exchanges and cooperation, and innovating institutional mechanisms. |
| June 2021 | 14th Five-Year Plan for the Integrated Development of Culture and Tourism in Tianjin | Tianjin Municipal Bureau of Culture and Tourism | Coordinate the advancement of cultural initiatives, cultural industries, and tourism resource development. Actively engage in the construction of the Grand Canal and the Great Wall National Cultural Parks. Additionally, conduct an in-depth exploration of the cultural heritage associated with the Tianjin Section of the Grand Canal, the Huangyaguan Great Wall, and the intangible cultural heritage of Tianjin Municipality. |
| July 2019 | Two-Year Action Plan for Tourism Development in Tianjin (2019-2020) | Tianjin Municipal Bureau of Culture and Tourism | We will coordinate the development of cultural initiatives, cultural industries, and tourism resources. We will actively engage in the construction of the Grand Canal and Great Wall National Cultural Parks, while thoroughly investigating the cultural heritage and intangible cultural heritage of the Tianjin section of the Grand Canal. |
| September 2021 | Plan for Strengthening Supervision of Tourism Service Quality and Improving Service Level | Hebei Provincial Department of Culture and Tourism | Take the initiative to align with national strategies by focusing on the coordinated development of the Beijing-Tianjin-Hebei region and the establishment of the Grand Canal National Cultural Park. Prioritize the cultivation of several world-class tourist attractions and resorts, including the Splendid Great Wall and the Charming Grand Canal. |
| August 2020 | Plan for the Integrated Development of Culture and Tourism in Shandong Province | Shandong Provincial Department of Culture and Tourism | The Grand Canal National Cultural Park (Shandong) will be developed within the cultural tourism demonstration zone of the Shandong Section of the Grand Canal. By employing the models of Culture + Tourism and Cultural Tourism +, innovative cultural tourism formats will be cultivated to generate new impetus for industrial development. |
| May 2021 | Notice on Issuing the Implementation Plan for Integrated Development of Culture and Tourism of the Shandong Section of the Grand Canal | Shandong Provincial Department of Culture and Tourism | Implement ten major initiatives, including the Grand Canal ecological environment protection and restoration project, the intangible cultural heritage preservation project, the cultural value exploration project, the creation and performance of high-quality literary and artistic works project, the revitalization of the cultural tourism industry project, the cultural tourism digitalization project, and the brand promotion initiative for Friendly Shandong · Shandong Canal Charm. |
| June 2021 | Plan for Integrated Development of Grand Canal Culture and Tourism in Jiangsu Province | Jiangsu Provincial Department of Culture and Tourism | Drawing upon the rich cultural heritage of the Jiangsu Section of the Grand Canal, this plan articulates the core values and spiritual meanings inherent in Canal culture. It seeks to reinterpret these elements through diverse contemporary artistic expressions and to enhance the visibility and recognition of Canal civilization through high-quality exhibitions and promotional efforts. |
| November 2021 | 14th Five-Year Plan for Cultural and Tourism Development of Jiangsu Province | Jiangsu Provincial Department of Culture and Tourism | Emphasize the integrated characteristics of "Water + Culture," with water serving as the vein and culture as the soul. This approach aims to fully showcase the beauty of "Water Rhyme Jiangsu." Additionally, it is essential to construct and operate the China Grand Canal Museum in Yangzhou at a high level. |
| April 2021 | Plan for Establishing 4A-level Tourist Attraction of Wujiang Grand Canal Cultural Tourism Scenic Area | People's Government of Pingwang Town, Wujiang District, Suzhou City | We aim to establish the core landscape of Pingwang Convergence of Four Rivers, recognized as one of the Ten Grand Canal Scenes in Suzhou. Our objective is to transform this site into an iconic representation of the Grand Canal Cultural Belt's development and the Jiangnan cultural brand. Additionally, we will carefully craft a contemporary interpretation of the Prosperous Canal Scroll. |
| September 2020 | Regulations of Zhejiang Province on the Protection of the Grand Canal World Cultural Heritage | Standing Committee of the People's Congress of Zhejiang Province | The Grand Canal heritage protection zone is categorized into heritage areas and buffer zones. Activities related to development and utilization outside the buffer zones must adhere to regulations concerning ecological environment protection, territorial space management, and other pertinent provisions. Furthermore, these activities should be compatible with the Grand Canal heritage and its historical characteristics. |
| February 2013 | Overall Tourism Planning of the Beijing-Hangzhou Grand Canal | Ministry of Culture and Tourism of the People's Republic of China | Enhance and develop the distinctive tourism features of the Beijing-Hangzhou Grand Canal, and provide new ideas and approaches for the innovative development of domestic cultural heritage tourism products. |
| May 2025 | Beijing Releases 2025 Action Plan for Protection, Inheritance and Utilization of the Grand Canal Culture | Beijing Municipal Development and Reform Commission | The initiative primarily encompasses a series of activities themed around the Grand Canal, the operation of the Beijing City Library with unique characteristics, the development of brands for Grand Canal art events, the organization of cultural and museum activities related to the Grand Canal, and the hosting of prestigious sports competitions centered on the Grand Canal. |

**Table S3.** Statistics on the Construction of Typical National/Prefectural-Municipal Cultural Parks of the BHGC.

| Canal Section | City | Year | Typical New / Major Upgraded Projects (Parks & Cultural/Sports Facilities) | Type & Remarks |
| --- | --- | --- | --- | --- |
| **Tonghuihe** | Beijing | 2021 | Beijing (Tongzhou) Grand Canal Cultural Tourism Scenic Area – North Zone (integrating the Randeng Pagoda and other heritage buildings) | Heritage restoration and scenic-area integration |
|  |  | 2022 | Grand Canal Cultural Tourism Scenic Area (unified application for national 5A-level scenic spot, acceptance check at year-end) | Integration of the “Three Temples & One Pagoda”, Canal Park, and the Grand Canal Forest Park |
|  |  | 2023 | **Grand Canal Museum of Beijing** (East Branch of the Capital Museum) | Entirely new; opened on December 27 |
|  |  | 2024 | Tongzhou Grand Canal Music Park (regular operation) | Newly built professional outdoor music venue |
| **North GC** | Tianjin | 2022 | Launch of the Wuqing Grand Canal Cultural & Ecological Development Belt (thematic upgrades for multiple parks) | Strengthened “Grand Canal” branding at North Canal Country Park and other sites |
| **South GC** | Langfang | 2018 | Xianghe Canal Cultural Park | Newly built; landmark park along the Xianghe section of the North Canal |
|  |  | 2023 | North Canal Cultural & Leisure Corridor (upgrades at multiple nodes) | Systematic construction of riverside greenways and pocket parks |
|  | Cangzhou | 2022 | **Cangzhou Grand Canal Garden Expo Park** (including the Grand Canal Intangible Cultural Heritage Exhibition Hall) | Entirely new; opened in August |
|  |  | 2023 | Baishi Park– Grand Canal Cultural Belt upgrade | Renovation, showcasing Cangzhou’s canal “Lion City” culture |
|  | Dezhou | 2023 | Dezhou Grand Canal Cultural Park | Newly built and opened |
|  | Liaocheng | 2021 | Liaocheng China Canal Culture Museum | Comprehensive upgrade of the old museum with redesigned exhibitions |
| **Weishanhu GC** | Jining | 2025 | Grand Canal Governor’s Office Ruins Park | Newly built; displays the history of the Canal Governor’s office |
|  | Zaozhuang | 2024 | Taierzhuang Grand Canal National Cultural Park | Cultural tourism integration hub |
| **Jiangnan GC** | Suqian | 2024 | Canal Bay Park (upgraded for sports and ecological integration) | Added Grand Canal-themed sports facilities and camping base |
|  | Huai’an | 2022 | Grand Canal “Hundred-Li Gallery” (opening of Qingjiangpu section and other nodes) | Integrating historical sites, green spaces, and slow-traffic systems |
|  | Yangzhou | 2020 | **Yangzhou Canal Grand Theatre** | A canal cultural landmark |
|  |  | 2021 | **Yangzhou China Grand Canal Museum** | Entirely new; a landmark museum |
|  | Zhenjiang | 2023 | Jingkou Gate Archaeological Site Exhibition Area-Grand Canal Cultural Belt upgrade | Outdoor display of the archaeological site combined with waterfront green space |
|  | Changzhou | 2022 | Grand Canal Industrial Heritage Exhibition Belt (Oil Tank Park and other nodes) | Industrial heritage transformed into canal-themed cultural and sports spaces |
|  | Wuxi | 2021 | Canal Art Park (comprehensive upgrade of the Canal Park) | Blending modern art, sports, and canal memory |
|  | Suzhou | 2017 | Suzhou Canal Sports Park (renovated and reopened) | Sports-themed Park integrated with canal cultural landscapes |
|  | Hangzhou | 2021 | **Hangzhou Grand Canal Asian Games Park** | Entirely new; Asian Games venue and urban park |
|  |  | 2021 | Hangzhou Grand Canal Music Park | Newly built; small professional venue for music events |
|  |  | 2024 | Grand Canal Hanggang Steelworks Park (partially opened) | Industrial heritage transformation, including cultural/sports, exhibition, and green spaces |
|  |  | 2025 | **China Beijing-Hangzhou Grand Canal Museum** | Entirely new; designed as a signature museum for the entire canal system |
|  | Jiaxing | 2022 | Grand Canal Digital “Poetry Road” e-Station (Nanhu Experience Center) | Digital culture experience center integrated into canal-side green space |

2. **Information on field survey sites and respondents in cities along the BHGC**

Our field survey was carried out in two phases: the first phase from April to June 2024 (covering the northern and middle reaches of the canal), and the second phase from September 2024 to January 2025 (covering the southern reach, Jiangnan reach and eastern Zhejiang extension of the canal). A total of 67 respondents were involved, including 14 industry experts and scholars, and 53 staff from sports, culture and tourism administrative departments of canal cities as well as scenic area operation practitioners. The survey forms included on-site field visits and structured, semi-structured and unstructured in-depth interviews, with each interview lasting 60–90 minutes. Detailed information is presented in the following table.

**Table S4.** Statistical of field survey sites and respondent information

| No. | Survey Site | Corresponding Canal | Survey Period | Number of Respondents | Key Survey Contents |
| --- | --- | --- | --- | --- | --- |
| 1 | Tongzhou District, Beijing | Tonghuihe | Apr-2024 | 4 | Field exploration of canal cultural venues and expert seminars |
| 2 | Wuqing District, Tianjin |  | Apr-2024 | 3 | On-site investigation of fitness trails along the canal, interviews on intangible cultural heritage folk sports programs, and exchanges with management staff |
| 3 | Cangzhou City, Hebei Province | North Canal | May-2024 | 3 | Investigation of martial arts intangible cultural heritage sports bases, field visits to canal sluice relics, and interviews with competent departments |
| 4 | Dezhou City, Shandong Province |  | May-2024 | 3 | Investigation of canal tea culture cultural tourism blocks and on-site survey of national fitness corridors |
| 5 | Liaocheng City, Shandong Province | South Canal | Jun-2024 | 6 | Census of sports, culture and tourism resources in the ancient canal city, interviews with university experts, and visits to water sports bases |
| 6 | Jining City, Shandong Province |  | Jun-2024 | 5 | Investigation of Weishan Lake dragon boat race venues and field exploration of the intangible cultural heritage of canal boatmen’s chants |
| 7 | Zaozhuang City, Shandong Province |  | Jun-2024 | 3 | On-site survey of riverside leisure sports belts and interviews with heads of cultural tourism projects |
| 8 | Xuzhou City, Jiangsu Province | Weishanhu | Sep-2024 | 5 | Field visits to canal heritage protection zones and seminars on outdoor sports cultural tourism projects |
| 9 | Suqian City, Jiangsu Province |  | Sep-2024 | 3 | Survey of riverside ecological fitness greenways and interviews on folk festival sports activities |
| 10 | Huai'an City, Jiangsu Province | Middle Canal & Li Canal | Oct-2024 | 4 | Investigation of water transport culture sports parks and joint thematic interviews with multiple departments |
| 11 | Yangzhou City, Jiangsu Province |  | Oct-2024 | 6 | Full-section reconnaissance of the Grand Canal national fitness corridor and immersive investigation of intangible cultural heritage folk sports |
| 12 | Zhenjiang City, Jiangsu Province |  | Oct-2024 | 3 | Field visits to riverside water sports venues and interviews on cultural tourism product development |
| 13 | Changzhou City, Jiangsu Province | Jiangnan Canal | Nov-2024 | 5 | Investigation of canal sports cultural and creative parks and in-depth exchanges with university sports tourism experts |
| 14 | Wuxi City, Jiangsu Province |  | Nov-2024 | 4 | Investigation of riverside marathon tracks and regular venues for dragon boat races |
| 15 | Suzhou City, Jiangsu Province |  | Nov-2024 | 6 | On-site recording of Dragon Boat boat boxing and dragon boat intangible cultural heritage programs, and survey of cultural tourism in ancient canal towns |
| 16 | Jiaxing City, Zhejiang Province |  | Dec-2024 | 3 | Visits to water town folk sports programs and investigation of riverside leisure trails |
| 17 | Hangzhou City, Zhejiang Province |  | Jan-2025 | 5 | Field survey of Asian Games canal sports parks and seminars with provincial and municipal competent departments of culture, tourism and sports |
| 18 | Ningbo City, Zhejiang Province | Eastern Zhejiang Canal Extension | Jan-2025 | 3 | Census of leisure sports resources in canal tributaries and interviews with cultural tourism operation practitioners |

3. **Interview outline information and methods for extraction, screening and classification of SWOT elements**

**3.1 Interview outline information**

Field investigations targeting various participants engaged in sports cultural tourism in canal cities and the corresponding interview outline information are shown below.

**(1) Interview Outline for Staff from Governments and Relevant Administrative Departments**

1) As a city along the BHGC, in your opinion, what characteristic sports cultural tourism projects are available for this section of the Canal?

2) Have you participated in or organized traditional sports activities along the BHGC? What is the current development status of such traditional sports?

3) What policies have been formulated to support the development of traditional sports along the BHGC?

4) In your view, how should governments, schools and civil organizations coordinate with one another to promote the sustainable development of sports cultural tourism along the BHGC?

5) Since the successful World Heritage inscription of the BHGC, what canal-themed cultural and sports supporting facilities have been built locally? What intangible cultural heritage projects are present here?

6) What substantial support and development dividends have the relevant policies issued by the central government and provincial/municipal governments brought to the integrated development of local SCT?

7) How does this city develop sports tourism routes themed on the BHGC? Are the transportation conditions for cross-regional collaboration well-established?

8) What direct competitive pressures does the BHGC’s characteristic sports-themed cultural tourism face?

9) For sports cultural tourism products in cities along the BHGC, how can we tackle the dilemma of weak brand recognition and insufficient appeal to tourists?

10) From a long-term development perspective, what is the core bottleneck that needs to be prioritized to realize the integrated development of sports cultural tourism along the BHGC?

**(2) Interview Outline for Experts and Scholars**

1) What are the cultural implications and historical significance of traditional canal sports?

2) How has the evolution of traditional canal sports initiatives changed since the implementation of reforms and opening-up policies?

3) Amid the amalgamation of canal sports cultural tourism, how can we achieve harmonized management across educational institutions, research establishments, associations, and instructional venues for traditional sports?

4) From your perspective, what is the symbiotic relationship in the development of traditional canal sports among educational institutions, society, and governmental bodies?

5) What obstacles hinder comprehensive coordination among diverse administrative entities?

6) What are the merits of local sports tourism resources, and how appealing are they to visitors?

7) What measures can be implemented to mitigate the environmental impact on traditional canal sports culture?

8) What are the classifications and manifestations of traditional canal sports activities, along with the prevailing issues and corresponding remedies?

9) What development approach do you propose for the future of traditional canal sports?

10) What factors influence the caliber of development in canal sports culture?

11) Have universities in your city established interdisciplinary majors focused on sports tourism?

12) Do the current sports tourism offerings reflect the cultural significance of the BHGC? What unique experiential projects are currently absent?

13) What recent changes have emerged in the disposable income of urban and rural residents along the canal, as well as in their consumption demands for SCT?

14) Have you been involved in the formulation of planning, project evaluation, or research initiatives related to the BHGC’s SCT? What practical experiences could serve as useful references?

15) From your perspective, what represents the greatest potential for academic research and industrial practice in the domestic field of the BHGC’s SCT at this time?

**(3) Interview Outline for Scenic Area Managers and Practitioners**

1) Do you have an affinity for traditional canal sports?

2) What traditional festival sports events are organized locally?

3) Which traditional canal sports have you personally participated in?

4) How are local traditional canal sports activities organized and executed?

5) Where can individuals engage in traditional canal sports?

6) What is the typical scale of participation in traditional canal sports cultural tourism activities?

7) From your perspective, what are the key differences between modern sports events and traditional canal sports?

8) What local traditional canal sports resources and associated tourism support facilities are available?

9) In your opinion, what factors influence the development of sports cultural tourism in canal cities?

10) How can the effective inheritance of traditional canal sports be achieved, in your view?

**3.2 Methods for extraction, screening and classification of SWOT elements**

**(1) Multi-source primitive elements through qualitative coding**

We code four kinds of original data to obtain first primitive items for SWOT analysis.

1) Field surveys data: We code records from field surveys and statistics from facilities in 18 canal cities between 2021-2025 to obtain physical resource endowments, operational failures at the site and infrastructure status.

2) Interviews data: Verbal transcripts of interviews with government personnel, experts, scholars, scenic area managers and practitioners. Text excerpts on industrial strengths, development challenges, policy benefits and market risks are identified.

3) Policy documents: 37 special policies issued by national, provincial governments and prefectural/municipal governments between 2012-2025 regarding BHGC cultural heritage, outdoor sports, sports cultural tourism are studied. Policy clauses on industrial support, park development and inter-regional collaboration are classified as opportunity elements.

4) Literature data: We reviewed 128 articles in Chinese and English on the BHGC’s SCT. The results of research accepted by the academic community on industrial weaknesses and external competition threats are considered as additional reference elements.

From this analysis, we have identified 86 primitive element statements, comprising 32 internal elements (strengths and weaknesses) and 54 external elements (opportunities and threats).

**(2) Initial screening: standardized categorization.**

Following the fundamental principles of the SWOT analysis framework, where Strengths and Weaknesses pertain to internal factors of the sector of the BHGC’s SCT, while Opportunities and Threats relate to external macro and market conditions, the team categorized and organized all initial data, removing duplications, redundancies, and overlaps, and consolidating synonymous elements.

Subsequently, 41 primary elements were identified, comprising 13 strengths, 10 weaknesses, 11 opportunities, and 7 threats.

**(3) Second-round screening expert consensus demonstration.**

A list of 41 candidate elements was shared with 14 experts comprising sports tourism administrators, university scholars, and Grand Canal heritage conservation practitioners. In the initial round, experts rated each element on a scale from 1 (completely irrelevant) to 5 (extremely representative). Elements scoring below 3.5 on average were excluded, and missing elements were added based on expert input. Subsequently, the revised list of 33 items from the first round was circulated again for scoring and consistency assessment. Elements with an expert consistency coefficient of ≥ 0.75 were retained as official SWOT elements. Disputed items were deliberated collectively through online seminars until a consensus was reached.

Through this procedure, we identified 20 formal core SWOT elements: 5 strengths, 4 weaknesses, 6 opportunities, and 5 threats. These elements serve as the foundation for the SWOT analysis presented in this paper.

**(4) Cross-validation of validity and classification calibration.**

All final SWOT elements were cross-checked with 3 types of data sources (field statistics, policy text, core viewpoints from interviews) to ensure that each element had evidence supported by at least 2 independent sources.

We also set standard classification criteria as follows: Strengths (S) are inherent favorable conditions for canal sports cultural tourism resources, industrial foundation and policy foundation; Strengths (W) are inherent constraints that hinder industry high-quality development; Opportunities (O) are external macro policies, market demand and cultural heritage construction dividends independent of industry supporting industrial growth; Threats (T) are external competitive pressure, market segmentation and talent constraints which can threaten industrial development.

All SWOT items in Section 3.2 follow the same screening and classification criteria.

4. **Supplementary ethical procedures for expert interviews**

All interview activities implemented standardized human research ethics management procedures. This research submitted the social science interview ethics self-declaration form to the Academic Ethics Committee of Liaocheng University, and the committee confirmed that this non-invasive expert consultation interview meets the criteria for human subject research exemption for social science studies.

This research belongs to non-invasive social science consultation interviews, does not involve physical sampling, physiological testing, psychological intervention or sensitive private information collection of participants; all respondents are industry managers, university scholars and public institution staff who voluntarily participate in industrial policy consultation, which conforms to the university’s social science interview ethics exemption criteria. Full ethical operation workflow during interviews is specified below:

(1) **Informed consent acquisition**

Prior to each interview, researchers presented participants with a written informed consent form outlining the research purpose, interview topics, recording guidelines, data handling procedures, anonymization techniques, and the participants' unrestricted right to withdraw without consequences. Prior to commencing interviews, all participants voluntarily signed the consent form; verbal consent was secured beforehand for remote telephone interviews and documented as supplementary proof.

(2) **Personal information anonymization & de-identification**

Following the interviews, all original audio recordings and verbatim transcripts underwent comprehensive de-identification procedures immediately. This process involved replacing all personal identifiers such as names, affiliations, contact details, specific geographical references, and any other identifiable information with anonymous codes (e.g., Expert 01, Local Culture Bureau Staff A). The manuscript, figures, tables, and supplementary materials do not contain any real individual identity information.

(3) **Information confidentiality and data protection rules**

Audio recordings, signed consent forms and original transcript files are stored in password-encrypted offline hard drives, only accessible to the two corresponding authors; all raw data will be permanently deleted 5 years after the completion of follow-up research;

All analysis content only extracts collective industrial viewpoints without quoting any individual’s unique identifiable remarks; cross-regional integrated statistical results are used in the paper to avoid tracing back to single interviewees;

Interview data is only used for this Grand Canal sports culture tourism research, and will not be shared, transmitted or used for other commercial or academic projects without additional written consent from participants.

(4) **Participation right protection**

All participants were informed they could refuse to answer any sensitive questions, suspend or terminate the interview at any stage, and request to delete partial or full recorded materials. No participants raised objections or requested data removal during the whole research process.
